# Wing Coupling in Bees and Wasps: From the Underlying Science to Bioinspired Engineering

**DOI:** 10.1002/advs.202004383

**Published:** 2021-06-03

**Authors:** Sepehr H. Eraghi, Arman Toofani, Ali Khaheshi, Mohammad Khorsandi, Abolfazl Darvizeh, Stanislav Gorb, Hamed Rajabi

**Affiliations:** ^1^ Faculty of Mechanical Engineering University of Guilan Rasht 4199613776 Iran; ^2^ Division of Mechanical Engineering Ahrar Institute of Technology and Higher Education Rasht 4193163591 Iran; ^3^ Functional Morphology and Biomechanics Institute of Zoology Kiel University Kiel 24118 Germany

**Keywords:** bee‐inspired joint, natural joint, asymmetry, biomechanics, functional diptery, hamuli, biomimetics

## Abstract

Wing‐to‐wing coupling mechanisms synchronize motions of insect wings and minimize their aerodynamic interference. Albeit they share the same function, their morphological traits appreciably vary across groups. Here the structure–material–function relationship of wing couplings of nine castes and species of Hymenoptera is investigated. It is shown that the springiness, robustness, and asymmetric behavior augment the functionality of the coupling by reducing stress concentrations and minimizing the impacts of excessive flight forces. A quantitative link is established between morphological variants of the coupling mechanisms and forces to which they are subjected. Inspired by the coupling mechanisms, a rotating‐sliding mechanical joint that withstands tension and compression and can also be locked/unlocked is fabricated. This is the first biomimetic research of this type that integrates approaches from biology and engineering.

## Graphical abstract

Although wing‐to‐wing coupling mechanisms have a common function, i.e., synchronizing motions of insect wings and preventing their aerodynamic interference, they are morphologically diverse. Quantitative links are established between the design of coupling mechanisms and forces to which they are subjected. The underlying design strategies are then used to develop a bioinspired mechanical joint for industrial and robotic applications.

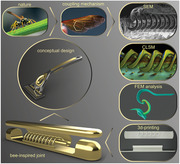

## 1 Introduction

**Figure 1:**
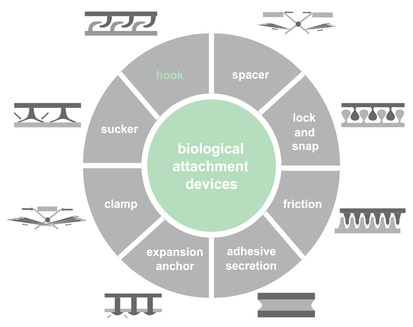
Biological attachment devices. Biological attachment devices can be subdivided into eight categories of **“**hook,” “sucker,” “clamp,” “expansion anchor,” “adhesive secretion,” “friction,” “lock and snap,” and “spacer.” Reproduced with permission.^[^ ^1^ ^]^ Copyright 2002, Springer Nature.

Biological attachment devices are ubiquitous in nature. They are primarily used for permanent or temporary attachment of different body parts of an animal to a substrate or to each other. There are eight types of attachment devices in nature, namely “hook,” “sucker,” “clamp,” “expansion anchor,” “adhesive secretion,” “friction,” “lock and snap,” and “spacer” (**Figure** **1**).^[^ ^1^ ^]^ Although all of them share a common function, i.e., attachment, only one of them works under high‐frequency cyclic loading, i.e., “hook.” Wing‐to‐wing coupling mechanisms, which mainly possess hook‐like or clamp‐like structures, exemplify such attachment devices, which are prevalent among some insect orders, including Hymenoptera,^[^ ^2^ ^]^ Hemiptera,^[^ ^3^, ^4^, ^5^ ^]^ Psocodea,^[^ ^6^, ^7^ ^]^ Lepidoptera,^[^ ^8^, ^9^ ^]^ and Trichoptera.^[^ ^10^ ^]^

In Hymenoptera, the elaborate coupling mechanism acts as a multifunctional joint. It couples a rolled membrane at the trailing edge of the forewing to a set of hook‐like structures at the leading edge of the hind wing during flight and uncouples them at rest.^[^ ^11^ ^]^ It also allows the relative displacement of fore and hind wings in flight, considering that they have different joints on the body and therefore different rotation axes. By synchronizing fore and hind wings, the coupling mechanism enables insects to attain more lift and better gliding performance.^[^ ^12^ ^]^ The disability of the coupling mechanism results in aerodynamic interference of the wings,^[^ ^13^ ^]^ reduces lift,^[^ ^13^ ^]^ and can even lead to flight inability.^[^ ^14^ ^]^

The insect order Hymenoptera, which includes bees, wasps, ants, and sawflies, represents one of the most diverse forms of life, with more than 153 000 identified species.^[^ ^15^ ^]^ As Hymenoptera comprise the overwhelming majority of pollinators, predators, as well as parasitoids, they play a vital role in almost all terrestrial ecosystems.^[^ ^15^ ^]^ Former studies have shown that the morphology of the coupling mechanisms significantly varies across different hymenopteran species, although they all serve the same function.^[^ ^2^ ^]^ While a few previous attempts have been made to investigate the presence of a correlation between flight range, bodyweight, wing length and the morphology of the coupling mechanism, a clear understanding of the biomechanical factors that underlie their diversity remains elusive.^[^ ^16^, ^17^, ^18^, ^19^ ^]^ To fill this gap in the literature, we present the first ever systematic investigation of the structure–material–function relationship of the coupling mechanisms in Hymenoptera. We hypothesize that the coupling mechanisms have undergone morphological adaptations to forces to which they are subjected. We selectively collected nine distinct castes and species of bees and wasps to cover a broad range of lifestyles, bodyweights, and load‐lifting capacities across Hymenoptera. We combined modern imaging techniques, including scanning electron microscopy (SEM), and confocal laser scanning microscopy (CLSM), with finite element (FE) method, computer‐aided design, and 3D printing to address the following unexplored questions:

1) How do morphological parameters vary within single coupling mechanisms?
2) How do morphological parameters vary among the coupling mechanisms of Hymenoptera?
3) Can morphological variations of the coupling mechanisms be explained by forces that they experience?
4) What are the morphological parameters that enable the coupling mechanisms to work under excessive flight forces without failure?
5) How could coupling mechanisms inspire the design of engineering joints?

## 2 Results

### 2.1 Morphology of the Coupling Mechanism of Worker Honeybees

**Figure 2:**
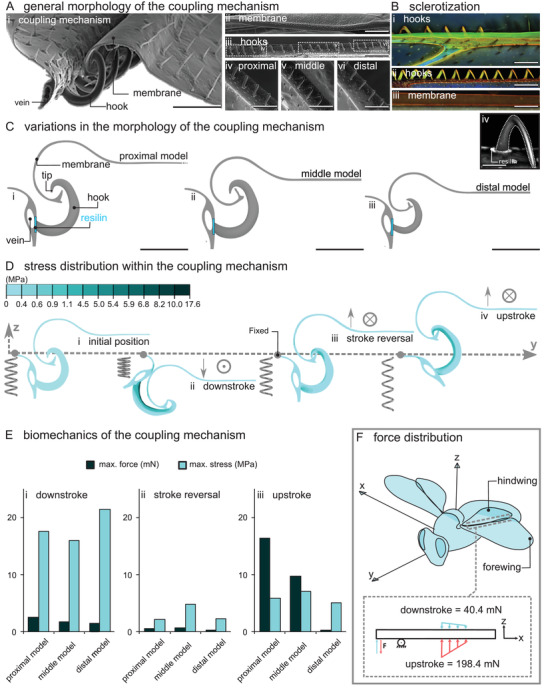
Morphology and biomechanics of the coupling mechanism of the worker honeybee *Apis mellifera*. A) SEM images of the coupling mechanism: Ai) hooks and membrane in the locked position, Aii) the rolled membrane, Aiii–vi) hooks. Aiv–vi) The proximal and middle hooks are out‐of‐plane and larger than distal hooks. B) CLSM images of the coupling mechanism: hooks and vein of the hind wing (ventral view in (Bi), dorsal view in (Bii)) and Biii) the membrane of the fore wing. Biv) A high‐contrast grayscale image shows the presence of the soft cuticle, likely resilin, at the junction of the hook and vein. C) Three models of the coupling mechanism: Ci) “proximal model,” Cii) “middle model,” and Ciii) “distal model.” D) Maximum principal stress developed within the coupling mechanism: Di) at the initial position, Dii) on the downstroke, Diii) during the stroke reversal, and Div) on the upstroke. Arrows and the symbols ⊙ and ⊗ show the displacement boundary condition applied to the membrane (⊙ for the “out of the page” motion and ⊗ for the “into the page” motion). The spring elements represent the springiness of the coupling mechanism: Dii) a compressed spring on the downstroke, Diii) a relaxed spring during the stroke reversal, and Div) a stretched spring on the upstroke. E) Comparison of the maximum stress within the models and the force they could resist without being unlocked: Ei) on the downstroke, Eii) during the stroke reversal, and Eiii) on the upstroke. F) Schematic representation of the force distribution along the coupling mechanism. The values show the estimated force that the coupling mechanism can withstand without being unlocked. Scale bars: Ai) 50 µm, Aii,Aiii) 200 µm, Aiv,Av,Avi,Bi,Bii,Biii) 100 µm, Biv) 30 µm, Ci,Cii,Ciii) 50 µm.

We started our research with an in‐depth investigation of the wing‐to‐wing coupling mechanism of the worker honeybee *Apis mellifera*. The coupling mechanism comprises three key structural elements: i) a rolled membrane at the trailing edge of the fore wing (**Figure** **2**Ai,ii), ii) a set of 20–22 minuscule hook‐like structures, called as hooks, that are situated in a row on the leading edge of the hind wing (Figure 2Ai,iii) and iii) a vein at the leading edge of the hind wing, in which the base of the hooks is embedded (Figure 2Ai). The proximal and middle hooks are almost identical in shape and size. They are relatively larger and more out‐of‐plane than the distal hooks. They possess conspicuous tips, which are absent in the distal hooks (Figure 2Aiv–vi). On the fore wing, the rolled membrane becomes narrower from the basal to the distal part (Figure 2Aii).

The CLSM images characterized a roughly equivalent level of sclerotization in the hooks of worker honeybees (Figure 2Bi,ii). The presence of the red and green autofluorescence in the rolled membrane, hooks, and vein, in which hooks are lodged, is an indication of a virtually sclerotized cuticle constituting the coupling mechanism (Figure 2Bi–iii). Nonetheless, the observation of a blue autofluorescence revealed the existence of a soft cuticle, likely resilin (according to the interpretation proposed by Michels and Gorb),^[^ ^20^ ^]^ at the junctions of the hooks and the vein (Figure 2Civ).

### 2.2 Biomechanics of the Coupling Mechanism of Worker Honeybees

We investigated the biomechanics of the coupling mechanism of the worker honeybees using FE analyses. We developed three models that represent the proximal, middle, and distal parts of the mechanism (Figure 2Ci–iii). The models allowed us to pinpoint the influence of the morphological variations of the hooks and membrane along the coupling mechanism on its biomechanical behavior. We simulated three potential interactions between the hooks and membrane in flight, including concurrent tension and slide during the i) downstroke, ii) stroke reversal, and iii) upstroke (Figure 2Dii–iv).

Considering the general resemblance of the overall deformation pattern and stress distribution of the “proximal model,” “middle model,” and “distal model,” here we only present the results of our simulations for the proximal model. The initial condition of the model and its deformations are illustrated in Figure 2D. The colors in the deformed models present the maximum principal stress. Our results suggest that the rolled membrane undergoes minute deformations, while the hook experiences perceptible deformations on both the down‐ and upstrokes. The vein, in contrast, exhibits a pronounced in‐plane revolution, up to ≈90°, from the downstroke to the upstroke. The stress level is higher along the interior part of the hook compared to the other regions, and is higher on the downstroke compared to the upstroke.

On the downstroke, the maximum stresses are equal to 17.6, 15.9, and 21.4 MPa within the “proximal model,” “middle model,” and “distal model,” respectively (Figure 2Ei). On the stroke reversal, the maximum stresses are relatively small and equal 2, 4.6, and 2.2 MPa for the “proximal model,” “middle model,” and “distal model,” respectively (Figure 2Eii). On the upstroke, the maximum stresses are rather close and equal to 5.5, 6.6, and 4.8 MPa for the “proximal model,” “middle model,” and “distal model,” respectively (Figure 2Eiii). Figure S1 in the Supporting Information illustrates the wing stroke trajectory of the worker honeybee in flight along with the corresponding deformation and stress distribution of the coupling mechanism. This visualizes how the coupling mechanism is likely to work in practice.

We also measured the forces that our models could withstand without being unlocked, so‐called unlocking forces (Figure 2E). Although the unlocking force on both the upstroke and downstroke represents a declining trend from the proximal to the distal hooks, this trend is steeper on the upstroke (downstroke: 2.5, 1.8, and 1.5 mN, stroke reversal: 0.5, 0.6, and 0.3 mN, upstroke: 15.5, 9.2, and 0.2 mN, Figure 2Ei–iii). It should be highlighted that the forces required to unlock the models are remarkably higher on the upstroke than on the downstroke, except for the distal model that resists a relatively small force on the upstroke. Figure 2F schematically illustrates the force distribution along the coupling mechanism on both the upstroke and downstroke. It shows that the load‐bearing capacity of the coupling mechanism on the upstroke is approximately five times higher than that on the downstroke (198.4 mN vs 40.4 mN, considering eight proximal, eight middle, and four distal hooks).

### 2.3 Morphological Variations of the Hooks across Species and Castes

**Figure 3:**
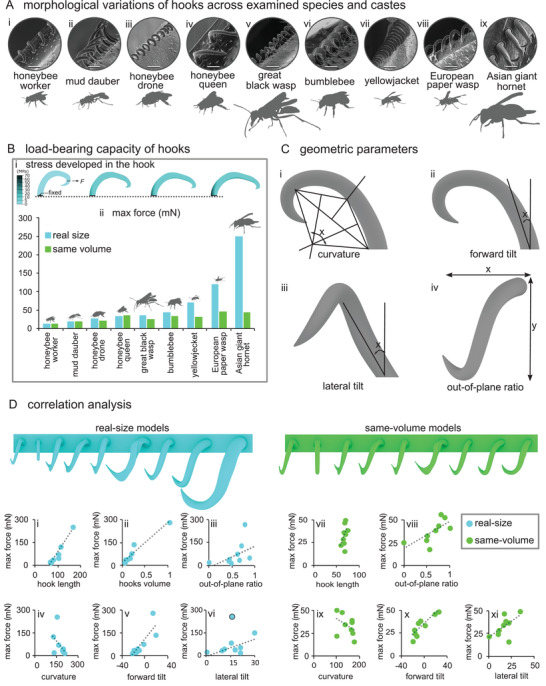
Comparison of the morphology of the hooks and their load‐bearing across different species and castes. A) SEM images of the hooks. B) Load‐bearing capacity of the hooks in tension. Bi) Maximum principal stress distribution in a representative hook of the Asian giant hornet. Bii) Load‐bearing capacity of the hooks in the “real‐size models” and “same‐volume models.” C) Geometric parameters of the hooks including Ci) curvature, Cii) forward tilt, Ciii) lateral tilt, and Civ) out‐of‐plane ratio. D) Correlation between the load‐bearing capacity of the hooks and morphologic/geometric parameters, including Di) hook length (load‐bearing capacity = −147.45 + 2.29 × length, *R^2^* = 0.95, *P* < 0.0001), Dii) hook volume (load‐bearing capacity = 16.48 + 244.13 × volume, *R^2^* = 0.94, *P* < 0.0001), Diii) out‐of‐plane ratio (load‐bearing capacity = −10.93 + 137.44 × out‐of‐plane ratio, *R^2^* = 0.24, *P* = 0.037), Div) curvature (load‐bearing capacity = 258.13 − 1.20 × curvature, *R^2^* = 0.22, *P* = 0.041), Dv) forward tilt (load‐bearing capacity = 114.52 + 4.62 × forward tilt, *R^2^* = 0.70, *P* = 0.001), and Dvi) lateral tilt (load‐bearing capacity = 5.42 + 2.82 × lateral tilt, *R^2^* = 0.50, *P* = 0.005), when the hooks are in their real size, and the correlation between the load‐bearing capacity of the hooks and morphologic/geometric parameters, including Di) hook length (load‐bearing capacity = −59.61 + 1.36 × length, *R^2^* = 0.23, *P* = 0.0001), Dii) out‐of‐plane ratio (load‐bearing capacity = 15.46–27.54 × out‐of‐plane ratio, *R^2^* = 0.48, *P* < 0.0001), Diii) curvature (load‐bearing capacity = 56.56–0.16 × curvature, *R^2^* = 0.19, *P* = 0.0001), Div) forward tilt (load‐bearing capacity = 38.31−0.69 × forward tilt, *R^2^* = 0.78, *P* < 0.0001), and Dv) lateral tilt (load‐bearing capacity = 20.32–0.77 × lateral tilt, *R^2^* = 0.34, *P* < 0.0001), when the hooks were scaled to have the same volume. Trend lines are linear regressions (gray dashed lines). The blue circle with dark outline in (Dvi) was excluded from linear regression analysis. Scale bars: Ai, Avi, Aix) 50 µm, Aii, Aiv) 30 µm, Aiii, Aviii) 80 µm, Av) 90 µm, Avii) 60 µm.

We studied the morphological variations of the coupling mechanisms, in particular their hooks, among nine species and castes of Hymenoptera, including worker honeybee, mud dauber wasp, drone honeybee, queen honeybee, great black wasp, bumblebee, yellowjacket wasp, European paper wasp, and Asian giant hornet. We found that morphological traits of the coupling mechanism, including length, shape, size, and number of hooks appreciably vary across the examined species (**Figure** **3**A).

The length of the coupling mechanism changes from a minimum of 0.64 mm in the yellowjacket wasp to a maximum of 2.06 mm in the great black wasp (**Table** **1**). Majority of the hooks in the coupling mechanism are curved, out‐of‐plane, and tapered. They have circular bases and conspicuous tips, except for the distal hooks that are mostly in‐plane and have no tips (Figure 3A). Despite the general similarities, there are obvious variations in the length, volume, curvature, tilt angle, and out‐of‐plane ratio of the hooks. Taking the hooks of worker honeybees as a “reference,” the proximal hooks of mud dauber wasps are elongated, in‐plane, and less curved (Figure 3Aii). The proximal and middle hooks of drone honeybees, and queen honeybees are somewhat bifurcated at the tip (Figure 3Aiii,iv). The hooks of black wasps are sharply turned at the tip (Figure 3Av). The hooks of bumblebees are more out‐of‐plane and have large tips (Figure 3Avi). The hooks of yellowjacket and European paper wasps are more out‐of‐plane and have relatively larger tips and are less tilted forward. Moreover, the hooks in the last two groups resemble each other except that those of paper wasps are more laterally tilted at the base (Figure 3Avii,viii). The tips of hooks in Asian giant hornets are comparatively large and more turned, similar to those of bumblebees (Figure 3Aix). In terms of the size, the smallest hook length is ≈66 µm and belongs to mud dauber wasps, whereas the largest is ≈168 µm and was found in Asian giant hornets (Table 1). The number of the hooks in each coupling mechanism also notably differs from a minimum of 18 in queen honeybees to a maximum of 53 in great black wasps (Table 1). More SEM and CLSM images of the coupling mechanisms of the studied castes and species are available in Figures S2 and S3 in the Supporting Information.

**Table 1:** Summary of the morphological and biomechanical data of the coupling mechanisms across examined species and castes. Mean values are given for the morphological data

Study groups	Insect body weight [mN]	Number of hooks	Hook length [µm]	Length of the coupling mechanism [mm]	Load‐bearing of hooks in “real‐size models” [mN]	Load‐bearing of hooks in “same‐size models” [mN]	Load‐bearing of the coupling mechanism^a)^ [N]
Worker honeybee	1.10	22	67.8	0.71	15.2	15.2	0.33
Mud dauber wasp	1.30	33	66.3	1.48	20.0	21.0	0.66
Drone honeybee	2.80	24	73.7	1.55	28.0	23.5	0.67
Queen honeybee	2.10	18	78.0	1.15	34.3	27.5	0.62
Great black wasp	4.90	53	80.0	2.06	36.0	32.6	1.91
Bumblebee	3.30	21	101.5	1.07	45.0	35.0	0.94
Yellowjacket wasp	0.75	26	102.5	0.64	71.8	37.0	1.87
European paper wasp	0.85	20	113.3	1.38	122.4	47.4	2.45
Asian giant hornet	5.00	25	168.0	1.28	251.7	44.8	6.29

^a)^ This was estimated by multiplying the load‐bearing capacity of a proximal hook in each coupling mechanism by the number of hooks in that mechanism.

### 2.4 Biomechanics of Hooks across Species and Castes

To investigate the influence of the hook design on its mechanical performance, we subjected our isolated hook models to tension. This test enabled us not only to measure how much force each hook can withstand, but also to understand how the stress patterns in the isolated hooks differ from those of the hooks that were tested in the coupling mechanism (Figure 3Bi). We also unearthed the geometric parameters that influence the load‐bearing capacity of the hooks including curvature, out‐of‐plane ratio, forward and lateral tilt angles. We calculated the curvature of the hook using the method used by Birn‐Jeffery.^[^ ^21^ ^]^ We defined the ratio of the width to the length of the hooks as their “out‐of‐plane ratio” (Figure 3C).

Figure 3D outlines the main morphological differences between the hooks in two scenarios: i) when hooks are in their real size and ii) when they have the same volume (Figure 3D). This comparison includes the proximal hooks only, as they are subjected to the highest flight forces. In the “real‐size models,” the load‐bearing capacity of the hooks considerably varies from 15.2 mN in the worker honeybee to 251.7 mN in the hornet. However, when we exclude the effect of the volume, the load‐bearing capacity of the hooks ranges from 15.2 mN in the worker honeybee to 47.4 mN in the paper wasp (Figure 3Bii, Table 1).

We found a strong positive correlation between the load‐bearing capacity of the hooks and their length (load‐bearing capacity = −147.45 + 2.29 × length, *R^2^* = 0.95, *P* < 0.0001), meaning that longer hooks withstand higher forces (Figure 3Di). Almost the same relationship applies to the load‐bearing of the hooks and their volume (load‐bearing capacity = 16.48 + 244.13 × volume, *R^2^* = 0.94, *P* < 0.0001) (Figure 3Dii). The load‐bearing capacity had a weak correlation with the out‐of‐plane ratio (load‐bearing capacity = −10.93 + 137.44 × out‐of‐plane ratio, *R^2^* = 0.24, *P* = 0.037) (Figure 3Diii). A weak inverse relationship was found between the load‐bearing of the hooks and their curvature (load‐bearing capacity = 258.13 − 1.20 × curvature, *R^2^* = 0.22, *P* = 0.041) (Figure 2Div). Figure 3Dv shows that the load‐bearing capacity strongly escalates as the hook forward tilt increases (load‐bearing capacity = 114.52 + 4.62 × forward tilt, *R^2^* = 0.70, *P* = 0.001). We found a moderate relationship between the load‐bearing of the hooks and their lateral tilt angle (load‐bearing capacity = 5.42 + 2.82 × lateral tilt, *R^2^* = 0.50, *P* = 0.005), only when the data of the hornet model were excluded (Figure 3Dvi).

For the “same‐volume models,” there was a weak correlation between the load‐bearing capacity of the hooks and the hook length (load‐bearing capacity = −59.61 + 1.36 × length, *R^2^* = 0.23, *P* = 0.0001) (Figure 3Dvii). The correlation analysis between the load‐bearing capacity of the hooks and their curvature and lateral tilt resulted in *R^2^* = 0.19 (load‐bearing capacity = 56.56 – 0.16 × curvature, *P* = 0.0001) and *R^2^* = 0.34 (load‐bearing capacity = 20.32 – 0.77 × lateral tilt, *P* < 0.0001), respectively, indicating the existence of weak correlations between the load‐bearing and the mentioned parameters. However, the correlation analysis between the load‐bearing capacity of the hooks and their out‐of‐plane ratio, and forward tilt resulted in *R^2^* = 0.48 (load‐bearing capacity = 15.46 – 27.54 × out‐of‐plane ratio, *P* < 0.0001) and *R^2^* = 0.78 (load‐bearing capacity = 38.31 – 0.69 × forward tilt, *P* < 0.0001), respectively, suggesting the existence of moderate and strong correlations between the load‐bearing and the mentioned criteria, respectively (Figure 3Diii–xi). This indicates that the out‐of‐plane ratio and forward tilt have relatively higher impacts on the load‐bearing capacity of hooks than the curvature and the lateral tilt, when the effect of size is excluded.

**Figure 4:**
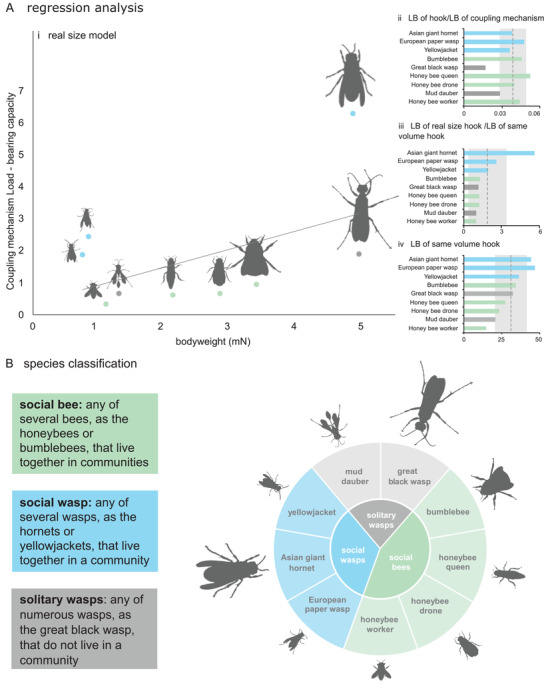
Relationship between the load‐bearing capacity of the coupling mechanism and insect lifestyle. Ai) Relationship between the load‐bearing of the coupling mechanism and the bodyweight of the examined species and castes (load‐bearing capacity of the coupling mechanism = 0.79–0.14 × body weight, *R^2^* = 0.29, *P* = 0.027). Trend line is a linear regression (gray solid line). Aii) Ratio of the load‐bearing capacity of the hooks to the load‐bearing capacity of the coupling mechanism. Aiii) Ratio of the load‐bearing capacity of the “real‐size models” to that of the “same‐volume models.” Aiv) Load‐bearing capacity of the “same‐volume models.” Dash lines and shaded areas in (Aii–iv) show the mean values and standard deviations of the data. LB, load‐bearing. B) Classification of the examined species and castes. They can be subdivided into three distinct groups of social wasps, solitary wasps, and social bees.

In **Figure** **4**Ai, we plotted the load‐bearing capacity of the coupling mechanisms versus insect bodyweights. A weak correlation exists between the two parameters (load‐bearing capacity of the coupling mechanism = 0.79 – 0.14 × body weight, *R^2^* = 0.29, *P* = 0.027). Explicitly, for the data points that fall below the regression line, the load‐bearing of the coupling mechanisms almost linearly increases by the increase of their bodyweight. However, the load‐bearing of the coupling mechanisms of the Asian giant hornet, paper wasp, and yellowjacket wasp lies above the regression line. This means that their coupling mechanisms can withstand higher forces than those of others with comparable bodyweight.

Figure 4Aii represents the load‐bearing capacity of the hooks as a fraction of the load‐bearing capacity of the coupling mechanism. The results show a noticeably smaller contribution of single hooks in the coupling mechanisms of the mud dauber and great black wasps to the overall load‐bearing capacity of those mechanisms. Figure 4Aiii shows the ratio of the load‐bearing of single hooks in the “real‐size models” to the “same‐volume models.” This ratio in the Asian giant hornet, European paper wasp, and yellowjacket is higher than those of the others and above the average ratio measured for the studied species. This indicates the larger contribution of the amount of the material used in the hooks of the three mentioned species to the load‐bearing of their hooks in comparison with the other studied groups. Figure 4Aiv compares the load‐bearing of isolated hooks when scaled to the same volume. Here again the hooks of the three species of the Asian giant hornet, European paper wasp and yellowjacket wasp have the three highest load‐bearing capacities. The lowest load bearing belongs to the scaled hook of the worker honeybee. This data can be used to compare the influence of the design on the load‐bearing of the hooks, excluding the size effect.

### 2.5 Bee‐Inspired Mechanical Joint

**Figure 5:**
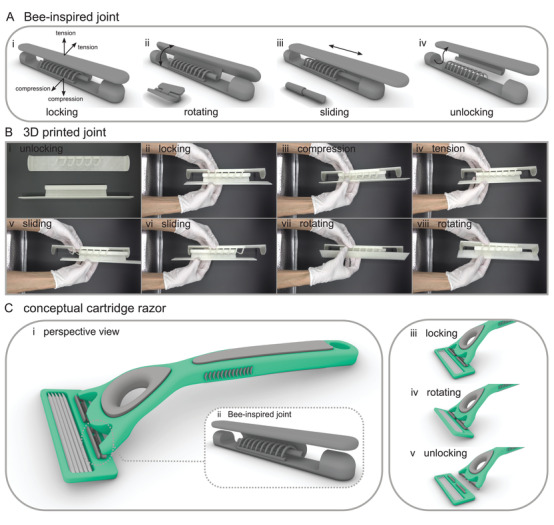
Bee‐inspired joint, 3D printing, testing, and application. A) Bee‐inspired joint with the ability to be Ai) locked and Aiv) unlocked, and it restricts Ai) motions in tension and compression but allows Aii) rotation and Aiii) sliding. B) 3D printing and testing. 3D printed joint Bi) in the unlocked position, Bii) in the locked position, Biii) under compression, Biv) under tension, Bv,Bvi) when sliding, Bvii,Bviii) when rotating. C) Conceptual cartridge razor. Ci,Cii) The design is supplemented with two bee‐inspired joints and is shown in Ciii) locking, Civ) rotating, and Cv) unlocking states.

Inspired by the wing coupling mechanism of Hymenoptera, we designed a mechanical joint capable of locking, rotating, sliding, and unlocking (Figure **5**Ai–iv). In the locking state, the joint can withstand both tension and compression, each in two orthogonal directions (Figure 5Ai). The joint can be easily unlocked by applying compression at a certain angle, as shown in Figure 5Aiv. Using 3D printing, we demonstrated the function of our joint in practice (Figure 5Bi–viii and Video S1, Supporting Information). The snapshots presented in Figure 5B show the motions of the 3D printed joint in a performed experiment. We designed a conceptual cartridge razor to display the use of our bee‐inspired joint in a real‐world application (Figure 5Ci–v and Video S2, Supporting Information).

## 3 Discussion

### 3.1 Biomechanics of the Coupling Mechanism

The remarkable flight capability of worker honeybees is primarily attributed to their two pairs of wings, which are coupled to each other in flight via a complicated coupling mechanism on each side. The wings not only withstand complex aerodynamic forces during flight, but they also undergo excessive mechanical stresses caused by accidental collisions.^[^ ^22^ ^]^ Surprisingly, the wings remain coupled during all loading scenarios owing to the presence of a row of hooks and a rolled membrane that are firmly interlocked. More importantly, the seemingly fragile hooks, which occupy a negligible fraction of the entire wing area (≈0.2%), continuously transfer forces from one wing to another. How are the hooks adapted to meet the functional demands, while preventing failure in various loading scenarios?

To address this question, we simulated the mechanical behavior of the coupling mechanism of worker honeybees during flight. We searched for design strategies that reduce the impact of excessive stresses. Surprisingly, we found that the maximum load that single hooks could withstand when situated in the coupling mechanism slightly exceeds that they could resist when tested in isolation. The encountered stresses were substantially lower within the hooks in the mechanism, in comparison with the isolated hooks (roughly 13 times). Moreover, in contrast to the isolated hooks, we found no stress concentration at the base of the hooks in the coupling mechanism, where we expected to see the highest bending moments. We suggest that this difference is the result of an increased level of compliance that is achieved when hooks are situated in the coupling mechanism. According to our numerical simulations, this compliance is accomplished through the presence of a soft patch at the hook base and a vein that can freely rotate within a certain range. The soft patch and the vein potentially act as a flexible joint, and thereby, mitigate the bending stresses at the hook base. This is an interesting finding, which suggests that the combination of some of the elements of the coupling mechanism produces a “springiness” effect, which stores the strain energy in the form of elastic deformation on a half‐stroke and releases it on the next half‐stroke, as seen in Video S3 in the Supporting Information. The spring elements in Figure 2 illustrate this mechanical behavior, as a compressed spring on the downstroke (Figure 2Dii), a relaxed spring in the middle of the stroke reversal (Figure 2Diii), and a stretched spring on the upstroke (Figure 2Div).

The coupling mechanism consists of hooks that have different forms and, therefore, resist different forces. The hooks situated at the proximal and middle parts of the coupling mechanism had much higher load‐bearing capacities than those at the distal part. The reason behind this difference can be found in the different forces to which the hooks are subjected, as their distance from the wing hinge increases. If we consider a constant muscle force at each flapping moment, we can write a rotational equilibrium between the muscle force and the forces applied to each hook along the coupling mechanism around the axis of rotation (i.e., wing hinge, Figure 2E,F). The equilibrium implies that as farther from the wing hinge the hook is, the lesser is the force that it experiences. This could explain why the load‐bearing capacity of the hooks decreases from the proximal hooks to the distal ones.

Another interesting finding here is the asymmetric behavior of the coupling mechanism on the up‐ and downstrokes. We found that the load‐bearing capacity of the coupling mechanism on the upstroke is a few times higher than that on the downstroke (approximately five times) (Figure 2F). This is surprising especially because we know that in contrast to some other insects, such as locusts, bees use both half‐strokes to produce lift.^[^ ^23^ ^]^ According to the literature data, however, this does not certainly imply that the forces generated on the up‐ and downstrokes are equal in the magnitude. In fact previous studies have shown that the bee wings produce higher forces on the upstroke than on the downstroke.^[^ ^24^ ^]^ Hence, the asymmetric load‐bearing of the coupling mechanism can be explained by the difference in the aerodynamic force generation on the up‐ and downstrokes.

Surprisingly, we found that the coupling mechanism of worker honey bees can withstand forces up to 180 times the insect bodyweight and 40 times the aerodynamic forces to which the wings may be subjected in flight. Why the coupling mechanism should be such robust? We propose that the answer lies in the wing collisions. Previous studies have shown a high risk of mechanical collisions between wings of flying insects with vegetation and other objects (see a review of the causes and consequences in Ref.^[^ ^22^ ^]^). In some cases, the number of such collisions reaches several thousands of times during the lifetime of an insect.^[^ ^25^ ^]^ Hence, the robustness of the coupling mechanism is a requirement for maintaining the link between the wings not only under typical flight forces, but also under unexpected loading scenarios.

### 3.2 Variations Across Examined Species and Castes

A diverse range of lifestyles has thrived within the order Hymenoptera.^[^ ^26^ ^]^ Hymenoptera represent the vast majority of socially organized insects, parasitoids, specialist predators, and herbivores.^[^ ^26^ ^]^ As a result, Hymenoptera have reached a high degree of complexity among all insect orders.^[^ ^26^ ^]^ Our SEM examinations demonstrated that the diversity and complexity also exist in the morphology of their wing‐to‐wing coupling mechanisms. The examined species in this study fall into three main categories of social wasps, solitary wasps, and social bees (Figure 4B). Despite their different lifestyles and bodyweights, they share one common feature: load‐lifting. The bodyweights of the examined species differ about an order of magnitude (Table 1), whereas their load‐lifting capacity varies from one‐fifth to almost nine times their bodyweight.^[^ ^27^, ^28^, ^29^, ^30^ ^]^ What are the strategies that enhance the load‐bearing capacity of the coupling mechanisms? Can the loads experienced by the coupling mechanisms explain their morphological variations among the different categories?

According to our results, three different strategies are served to improve the load‐bearing capacity of the coupling mechanisms of our examined species and castes: i) increasing the number of hooks, ii) enlarging hooks, and iii) improving the mechanical design of hooks. We found that solitary wasps, including black wasp and mud dauber, have used the first strategy, i.e., increasing hook number; Single hooks in this group had the lowest contribution to the overall load‐bearing capacity of the coupling mechanism among others (Figure 4Aii). Social wasps, in contrast, have mainly utilized the second strategy, i.e., enlarging the hooks. The examined members of this group, in particular Asian giant hornet, showed the highest ratios of the hook load‐bearing capacity in the real size to the scaled size (Figure 4Aiii). Social wasps are the only group that all its members, examined in this study, have employed the third strategy, i.e., hook design; When modeled in the same volume, hooks of social wasps showed higher load‐bearing than those of other groups (Figure 4Aiv). This suggests that, due to their design, hooks of the social wasps are more efficient than hooks of other groups. Future research should focus on a wider range of hymenopteran species to verify the generality of this finding.

Social wasps including yellowjackets, paper wasps, and hornets are predators and scavengers.^[^ ^31^ ^]^ These species transport their preys and, therefore, they had to be adapted to fly with heavy loads. This could explain our computational results indicating that the coupling mechanisms in this group have noticeably higher load‐bearing capacity than those of the other two groups. Solitary wasps, including mud dauber and black wasp are predators too. However, the ratio of the bodyweight of their preys to their own weight is far lower than that in social wasps (0.25–1.5 in solitary wasps, 0.5–9.0 in social wasps, according to refs. ^[^ ^24^ ^]^ and ^[^ ^29^ ^]^). This ratio is comparatively lower in social bees, such as honeybees and bumblebees, in which workers transport pollen and nectar. Worker honeybees carry loads 0.2–0.35 times their bodyweight.^[^ ^28^ ^]^ This ratio ranges from 0.23 to 0.91 in worker bumblebees.^[^ ^30^ ^]^ This data can explain the morphological variations of the coupling mechanisms across different groups, especially the more efficient design of the hooks in the coupling mechanisms of social wasps that has provided them with a noticeably higher load‐bearing capacity.

What design parameters have enhanced the load‐bearing capacity of the hooks? Our data obtained from the models that had the same volume suggested that the out‐of‐plane ratio and forward tilt angle are the most influential design parameters. This is in contrast to industrial joints that are mostly in‐plane, due to ease of manufacture. In an out‐of‐plane hook, the bending moments are transferred to more than only one axis, if compared with an in‐plane hook with the same length. This could reduce the bending stress at the hook base. The forward tilt, in contrast, changes the loading mode from bending to tension, which is often a less critical loading condition.

In addition to the investigation of the biomechanics of wing coupling mechanisms, here we aimed to use similar design strategies to develop an engineering joint with enhanced features, in comparison to the conventional joints. The most commonly used conventional joints in many daily applications are rigid, prismatic, revolute, cylindrical, spherical, and planer, each of which serves a single function. Our bee‐inspired joint not only combines the advantages of two existing engineering joints, i.e., revolute and cylindrical joints, but is also capable of resisting tension and compression in two orthogonal directions. In contrast to the conventional joints, without a compromise in its load‐bearing and robustness, our bee‐inspired joint can be simply disassembled. Using an example of a cartridge razor, we showed that our bioinspired design can be used in a real‐life application and transferred into a marketable technology. Our joint can be particularly used in robotic and industrial applications, where the easy and quick replacement of components is of importance.

We summarized our results and findings in a short video (Video S4, Supporting Information, https://onlinelibrary.wiley.com/page/journal/15214095/homepage/video-abstract-gallery.html).

## 4 Experimental Section

### Ethics

Processes involving animals were carried out in compliance with the authors’ institutional guidelines.

### Specimens

All species and castes, including the worker, drone, and queen honey bee *Apis mellifera*, the bumblebee *Bombus*, the mud dauber *Sceliphron caementarium*, the European paper wasp *Polistes dominula*, the yellowjacket *Vespula germanica*, the black wasp *Sphex pensylvanicus*, and the Asian giant hornet *Vespa mandarinia* were collected in Kumeleh‐Iran (37°9′26.7“N 50°10′24.1”E) during summer 2019. The specimens were euthanized by deep‐freezing and retained in 75% ethanol.

In this study, it was attempted to reduce the number of examined specimens as much as possible. For this purpose, the use of insect specimens was limited to morphological studies only and mechanical testing was replaced by finite element simulations. In order to ensure that the comparative results on the coupling mechanisms of the castes and species are not affected by limited number of examined specimens, a relatively larger number of worker honeybees (*n* = 15 for worker honeybees and *n* < 3 for other species and castes) were specifically collected. The results showed that morphological variations among individuals of a certain cast or species are negligible in comparison to those among the studied castes and species.

### Scanning Electron Microscopy

Fore and hind wings were removed from the collected insects. Before SEM, they were air‐dried, positioned on SEM stubs and sputter coated with a 7 nm thick gold layer in a sputter‐coater (Denton Desk II, Denton Vacuum, Moorestown, NJ). In total, 15 wing specimens were imaged using a TESCAN MIRA3 field emission SEM (TESCAN, Brno, Czech Republic) at 15 kV.

### Confocal Laser Scanning Microscopy

Hind wings were cut off and embedded in glycerine (≥99.5%, Carl Roth GmbH and Co. KG, Karlsruhe, Germany) between a glass slide and a cover slip. The specimens were then visualized with a Zeiss LSM 700 microscope (Carl Zeiss Microscopy, Jena, Germany) equipped with four stable solid‐state lasers with wavelengths of 405, 488, 555, 639 nm. The obtained images were used to determine the level of the sclerotization of the hooks, based on the autofluorescence of their materials: i) when excited with a stable solid‐state laser with a wavelength of 405 nm and visualized using a bandpass emission filter transmitting 420–480 nm, the soft nonsclerotized cuticle emits blue light; ii) when excited with a stable solid‐state laser with a wavelength of 488 nm and visualized using a longpass emission filter transmitting ≥490 nm, the less‐sclerotized cuticle emits green light; iii) when excited with stable solid‐state lasers with wavelengths of 555 and 639 nm and visualized using longpass emission filters transmitting ≥560 and ≥640 nm, respectively, the sclerotized cuticle emits red light.^[^ ^20^ ^]^ In total, seven wings were examined.

### Finite Element Modeling and Simulation

The computer‐aided design software Rhinoceros 6 was used to develop three 3D models that represent the coupling mechanism of the worker honeybee at proximal, middle, and distal parts (Figure 2Ci–iii). The models are called as the “proximal model,” “middle model,” and “distal model.” Each model consists of a hook, a vein, a resilin patch, and a rolled membrane (Figure 2Ci). Whereas the size of the rolling membrane decreases from the “proximal model” to the “distal model,” the size of the hooks is the same in the “proximal model” and “middle model” and becomes smaller in the “distal model.” The dimensions of the models were set to be equal to the average values of multiple measurements on the obtained SEM images.

The “proximal model” was designed to have a curved, out‐of‐plane, and tapered hook with a conspicuous tip, akin to a real hook in the proximal part of the coupling mechanism. The proximal model had a rolled membrane as a curved fold, like that of the worker fore wing (Figure 2Ci). The vein was modeled as a 90° fold at the edge of the leading edge of the hind wing, where the hooks are situated. For the “middle model” all mentioned features are the same except for the membrane, which is slightly more curved and narrower than the “proximal model” (Figure 2Cii). For the “distal model,” the hook size and membrane size were further decreased and the hook tip was removed (Figure 2Ciii).

The material property of the hook, vein, and membrane was presumed to be as that of the sclerotized cuticle: an elastic modulus of 6.8 Gpa,^[^ ^32^ ^]^ a Poisson's ratio of 0.3, and a density of 1200 kg m^−3^.^[^ ^34^, ^35^ ^]^ The effect of the soft cuticle, at the base of the hooks was simulated by modeling a soft patch at the junction of hook and vein. The soft patch had an elastic modulus of 700 MPa,^[^ ^33^ ^]^ a Poisson's ratio of 0.48,^[^ ^36^ ^]^ and a density of 1000 kg m^−3^.^[^ ^35^ ^]^ Comparison of the results obtained from the three models enabled to assess the influence of the morphological variations within the coupling system on its mechanical behavior.

In the next step, representative models of isolated hooks of the collected castes and species were developed (Figure 3D). The models were developed so that they represent the morphology of the hooks visualized in the SEM images. In total, nine hooks, called as the “real‐size models,” were modeled. The models enabled to carry out an in‐depth investigation to estimate the maximal force that hooks of each coupling mechanism could bear before breakage and to find a correlation between the load‐bearing capacity of the hooks and the forces experienced by them during insect lifespan. The same material properties as those mentioned in the previous section were used for the developed models. Consideration of the same properties for the hooks of the coupling mechanisms of the different insects seems to be a reasonable assumption. This is because cuticles of the hooks appear to be almost equally sclerotized in the examined species and castes (see the yellowish color of the hooks in Figure S3, Supporting Information). To better understand the impact of the hook morphology on its mechanical performance, the simulations were repeated for the hook models that were scaled to have the same volume. The new set of models was called as the “same‐volume models.”

To perform the numerical analysis, the models were imported into the ABAQUS FE software package (v.6.14; Simulia, Providence, RI). The general purpose eight‐node brick elements with reduced integration (C3D8R) were used with hourglass control, distortion control, and second‐order accuracy to mesh the models. Whereas using the reduced integration scheme decreased the computational runtime, the hourglass control, and distortion control prevented mesh distortions. For the soft cuticle at the hook base, comparatively smaller mesh size was used than that used for the rest parts of the models. This was because this part experienced large deformations under loading.

The ABAQUS explicit solver was used to simulate the mechanical behavior of the developed models. Three potential mechanical interactions between the components of the coupling mechanism were simulated: a simultaneous tension and slide on the i) upstroke, ii) downstroke, and during iii) stroke reversals. In all simulations, the fore wing and its rolled membrane were assumed to play the active role. In contrast, the hind wing vein and its hook were set to have no active motion, rather being passively deformed by the membrane. Moreover, the vein was fixed at its junction with the hind wing but was allowed to have a free in‐plane rotation (Figure 2D and Figure S4, Supporting Information). A “general contact algorithm” was used to define the interactions between the hook, the vein, and the rolled membrane. The friction coefficient between the contact surfaces was considered to be small and equal to 0.05.^[^ ^37^ ^]^ To simulate the downstroke, by applying a displacement boundary condition, the membrane was moved downward toward the hook while sliding them with respect to each other. To simulate the upstroke, the applied displacements were reversed. The change from the downstroke to the upstroke was defined as the stroke reversal. The magnitude of displacements in each loading scenario was set to be just smaller than the displacement above which the hook and the membrane would be unlocked.

After testing the hook and membrane models, the isolated “real hook models” were subjected to an upward tension, to simulate the loading on the upstroke. Before loading, all degrees of freedoms were fixed at the hook base. The loading was continued up to failure, which was predicted using the maximum principal stress theory. According to this theory, failure initiates when the stress within any element of the models reaches the strength of the material from which the models are made. The strength was considered to be equal to the strength of the sclerotized cuticle strength, i.e., 72 MPa.^[^ ^38^ ^]^ This simulation allowed to calculate the force required to break the “real size models” and compare it among the examined species and castes. The same procedure was applied to the “same volume model” to measure. This allowed to unearth the design parameters that have improved the load‐bearing of the hooks. For every simulation, a mesh convergence analysis was executed to eliminate the influence of the element size on the results. All the stress values reported in this research are the maximum principal stresses. Finally, Pearson correlation analysis was used to test the presence of a correlation between the load‐bearing of the hooks and their design parameters, including hook length, hook volume, curvature, out‐of‐plane ratio, forward tilt angle, lateral tilt angle of the hook base (Figure 3C).

### A Bee‐Inspired Joint: Design, Modeling, and 3D Printing

Using the computer‐aided design software Rhinoceros 6, a 3D model of a bee‐inspired joint was designed and developed. The model consisted of two parts that represent hooks and membrane in the real coupling mechanism (Figure 5A and Video S1, Supporting Information). To examine the function of the joint in practice, the design was fabricated using 3D printing. For this purpose, the 3D printer Prusa i3 MK3 (Prusa Research, Prague, Czech Republic) and polylactic acid filament (Amolen Firefly filament, filament diameter: 1.75 mm, printing temperature: 200–220 °C, Prusa Research) were employed. The bee‐inspired joint was then used in the conceptual design of a cartridge razor (Figure 5C and Video S2, Supporting Information).

### Statistical Analysis

Linear regressions were used to find the association between the dependent variables (e.g., load‐bearing capacity of the hooks and coupling mechanism) and independent variables (e.g., hook length, hook curvature, etc.). The significance of regression models was tested using the analysis of variance. All statistical analyses were carried out in Sigmaplot v.12.5 (Systat Software, San Jose, CA).

## Conflict of Interest

The authors declare no conflict of interest.

## Author Contributions

S.H.E. and A.T. contributed equally to this work. The author contribution is as follows: conceptualization: S.H.E., A.T., H.R., A.D., S.G.; supervision: A.D., H.R.; formal analysis: S.H.E., A.T., H.R.; investigation‐modeling and simulation: S.H.E., A.T.; investigation‐SEM: M.K., S.H.E., A.T.; investigation‐CLSM: H.R.; investigation‐3D printing: A.K.; project administration: H.R.; resources: A.D., S.G., M.K.; methodology: S.H.E., A.T., H.R.; validation: S.H.E., A.T., H.R.; visualization: A.T., S.H.E., A.K., H.R.; writing‐original draft preparation: S.H.E., H.R.; writing‐review and editing: S.H.E., A.T., A.K., M.K., A.D., S.G., H.R.

## Supporting information

Supporting Information

Supplemental Video 1

Supplemental Video 2

Supplemental Video 3

Supplemental Video 4

## Data Availability

The data that support the findings of this study are available from the corresponding author upon reasonable request.
