# Supplementary material for: Wing Coupling in Bees and Wasps: From the Underlying Science to Bioinspired Engineering
Source: Adv Sci (Weinh). 2021 Jun 3;8(16):2004383. doi: 10.1002/advs.202004383 (PMC8373159; doi:10.1002/advs.202004383)
Supplement: Supplementary file 1 — Supporting Information [file ADVS-8-2004383-s003.pdf]

© 2021 Wiley-VCH GmbH

ADVANCED SCIENCE
Open Access

Supporting Information

for *Adv. Sci.*, DOI: 10.1002/advs.202004383

Wing coupling in bees and wasps: From the underlying science to bioinspired engineering

*Sepehr H. Eraghi, Arman Toofani, Ali Khaheshi, Mohammad Khorsandi, Abolfazl Darvizeh, Stanislav Gorb and Hamed Rajabi**

Supporting Information

Wing coupling in bees and wasps: From the underlying science to bioinspired engineering

Sepehr H. Eraghi[1,2†], Arman Toofani[1,2†], Ali Khaheshi[3], Mohammad Khorsandi[1,2], Abolfazl Darvizeh[1,2], Stanislav Gorb[3] and Hamed Rajabi[3]*

[1] Faculty of Mechanical Engineering, University of Guilan, Rasht, Iran
[2] *Ahrar* Institute of Technology and Higher Education, Rasht, Iran
[3] Functional Morphology and Biomechanics, Institute of Zoology, Kiel University, Kiel, Germany

† These authors have contributed equally to this work
* Corresponding author email address: hrajabi@zoologie.uni-kiel.de; harajabi@hotmail.com

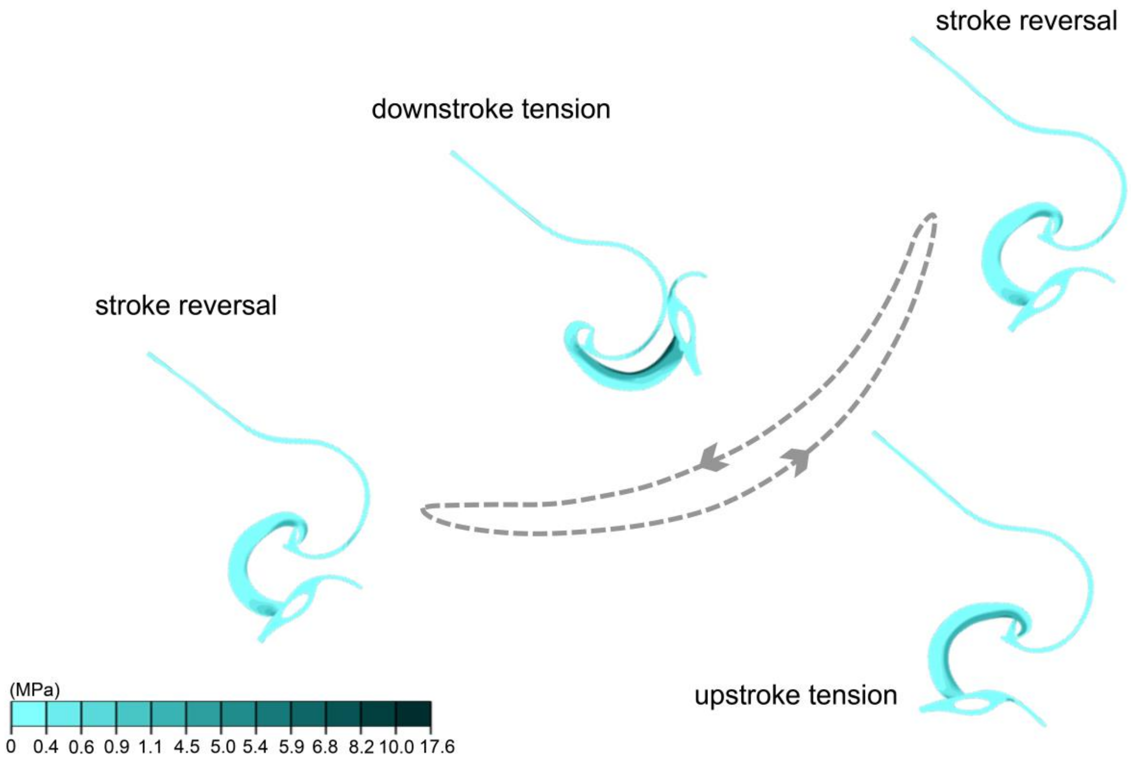

Figure S1. Orientation of the coupling mechanism during flight. Visualization of deformations and stress distributions during the (i) downstroke (ii) stroke reversal and (iii) upstroke. Dashed curve shows the wing stroke trajectory of the worker honeybee.

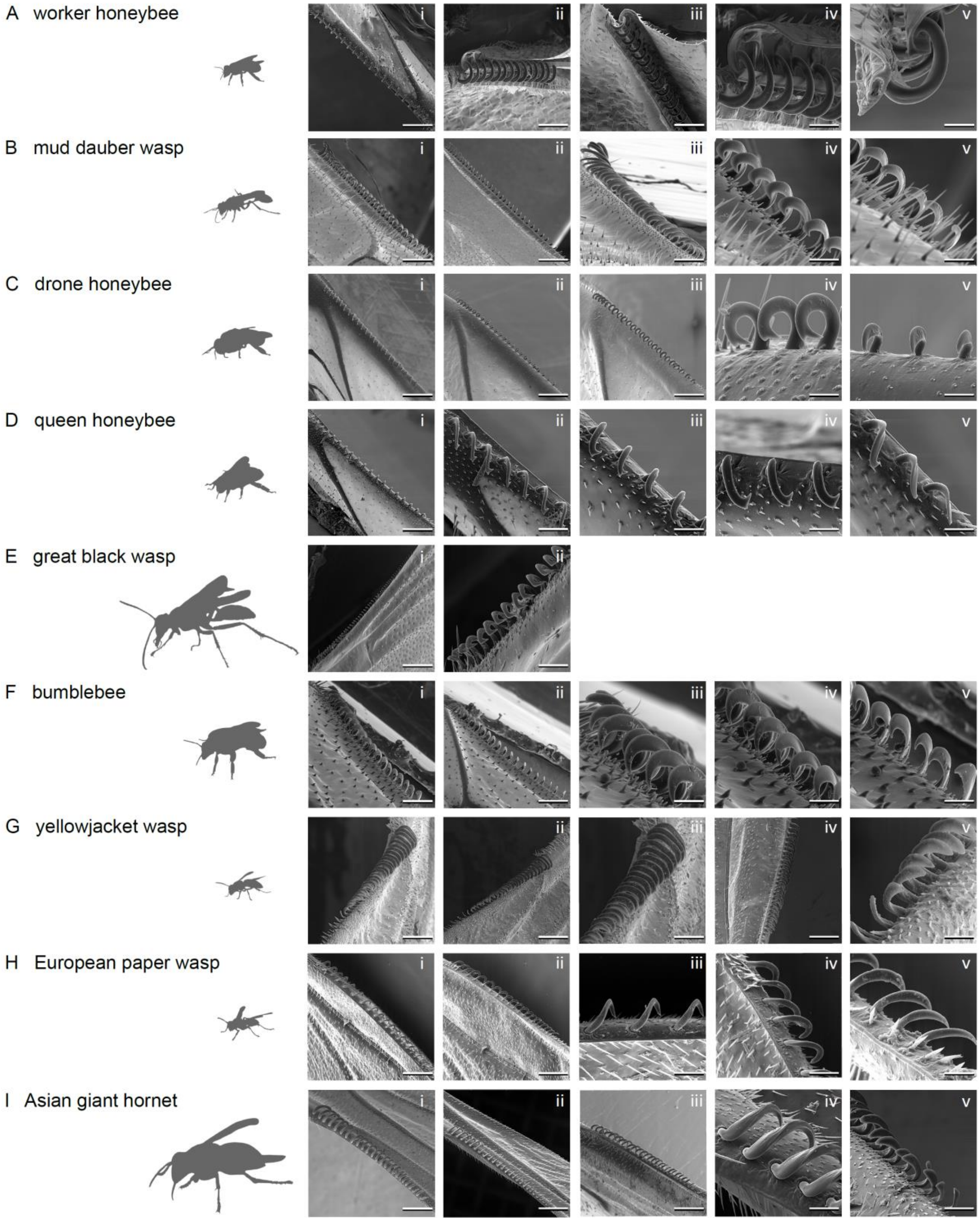

Figure S2. Morphology of the coupling mechanism and hooks across examined castes and species. (A) SEM images of the coupling mechanism of a worker honey bee. Hooks (Ai), hooks and membrane in the locked position (Aii-v). (B) Hooks of a mud dauber wasp (Bi-iii), proximal hooks (Biv), distal hooks

(Bv). (C) Hooks of a drone honeybee (Ci-iii), proximal hooks (Civ), distal hooks (Cv). (D) Hooks of a queen honeybee (Di), proximal hooks (Dii), middle hooks (Diii, iv), distal hooks (Dv). (E) Hooks of a Great black wasp (Ei), proximal hooks (Eii). (F) Hooks of a bumblebee (Fi, ii), proximal hooks (Fiii), middle hooks (Fiv), distal hooks (Fv). (G) Hooks of a yellowjacket wasp (Gi-iv), distal hooks (Gv). (H) Hooks of a European paper wasp (Hi, ii), proximal hooks (Hiii, iv), distal hooks (Hv). (I) Hooks of an Asian giant hornet (Ii-iii), proximal hooks (Iiv), distal hooks (Iv). Scale bars: 300 µm (Ei, Iii), 280 µm (Ci, ii), 220 µm (Ii, iii), 200 µm (Ai, Bii, Ciii, Di, Fii, Gii, Hi, Hii), 180 µm (Fi), 150 µm (Bi), 130 µm (Giv), 120 µm (Gi), 100 µm (Aiii, Eii), 90 µm (Aii), 80 µm (Ciii, Gii, Hiv), 75 µm (Giii), 60 µm (Diii, Hiii, Iiv), 50 µm (Biv, Dv, Fiv, Fv, Iv), 40 µm (Div, Fiii), 35 µm (Cv), 30 µm (Aiv, Civ, Gv, Hv).

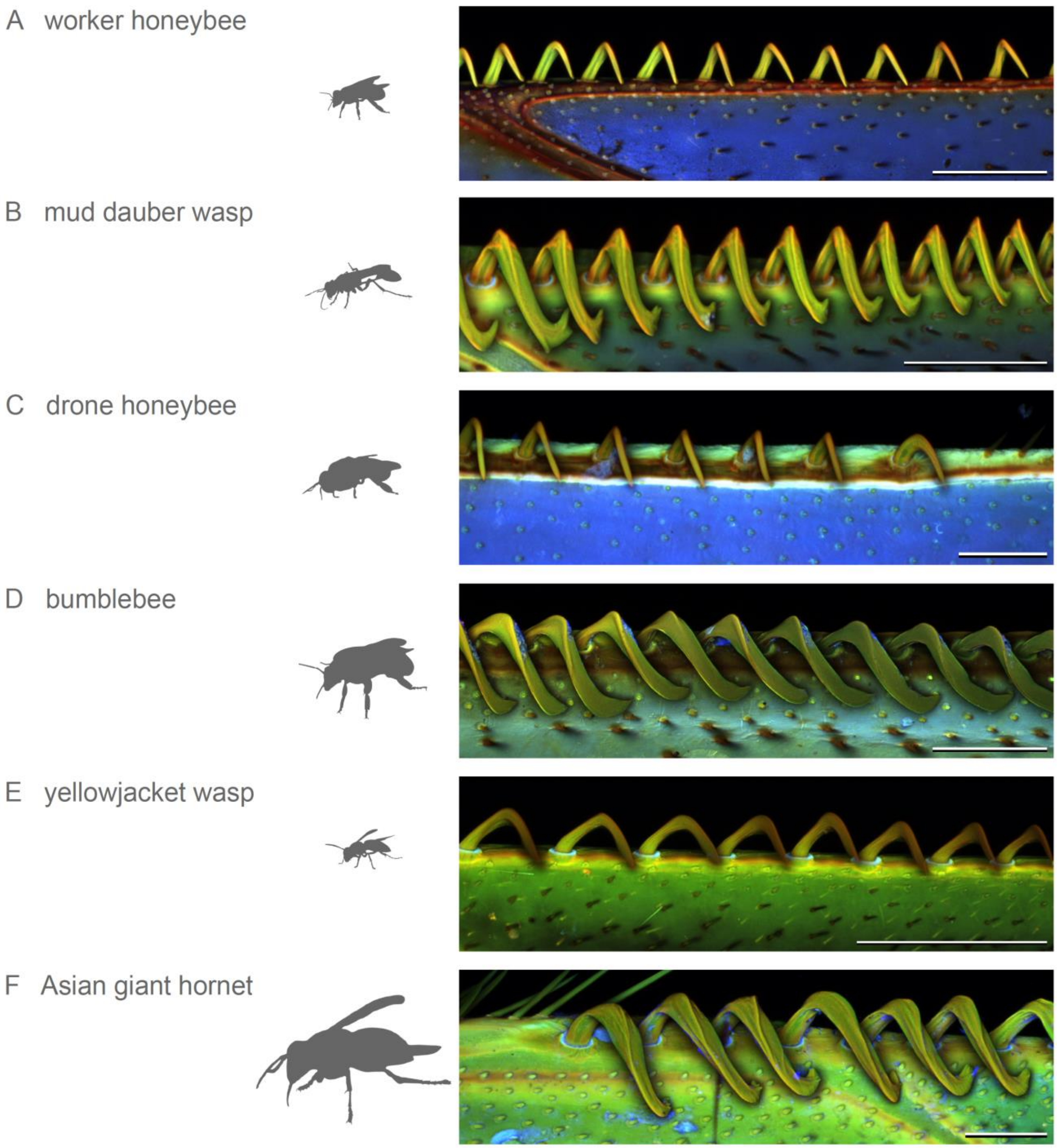

Figure S3. Sclerotization of the hooks. CLSM images of hooks of (A) worker honeybee, (B) mud dauber wasp, (C) drone honeybee, (D) bumblebee, (E) yellowjacket wasp, and (F) Asian giant hornet. Hooks appeared to be almost equally sclerotized. Scale bars: 100 µm.

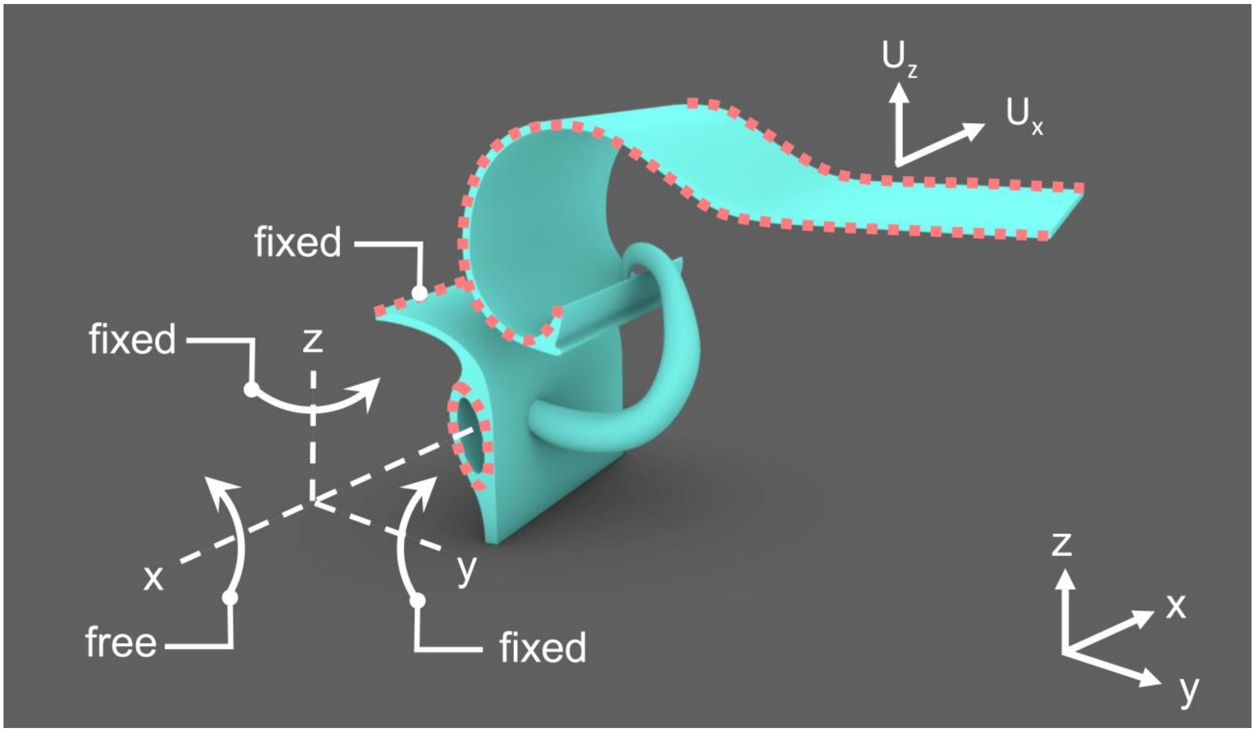

Figure S4. Boundary conditions in FE simulations. Vein was fixed at its base, but allowed to rotate about the x axis. Membrane was subjected to displacements in the positive x and z directions on the upstroke and in the negative x and z directions on the downstroke.
